# Phenolic and Terpenic Composition of *Salvia guaranitica* (Lamiaceae) Hydroethanolic Extract and Cardioprotective, Intestinal, and Anxiolytic Effects

**DOI:** 10.3390/plants15142182

**Published:** 2026-07-16

**Authors:** Soledad I. Matera, Ignacio Ceccato, María V. Piersante, Macarena Beteluz Majo, María L. Flores, Osvaldo L. Córdoba, Rocío Castilla, María I. Ragone, Alicia E. Consolini

**Affiliations:** 1 Laboratorio de Farmacología Experimental y Energética Cardíaca (LaFEyEC)-Cátedra de Farmacología, Depto Ciencias Biológicas, Facultad de Ciencias Exactas, Universidad Nacional de La Plata (UNLP), 47 y 115, La Plata 1900, Buenos Aires, Argentina; smatera@biol.unlp.edu.ar (S.I.M.); ceccatoignacio@gmail.com (I.C.); victoriapiersante@gmail.com (M.V.P.); macbeteluz@gmail.com (M.B.M.); 2 Grupo de Investigación en Química, Bioactividad y Metabolismo de Recursos Naturales Patagónicos y Área de Análisis Instrumental-Laboratorio de CromatografÍa e Instrumental (AAI-LACROMI), Centro Regional de Investigación y Desarrollo Científico Tecnológico (CRIDECIT), Facultad de Ciencias Naturales y Ciencias de la Salud, Universidad Nacional de la Patagonia San Juan Bosco, Km 4, s/N°, Comodoro Rivadavia 9000, Chubut, Argentina; mlujanflo@gmail.com (M.L.F.); okylola@gmail.com (O.L.C.); 3 Instituto Alberto C. Taquini de Investigaciones en Medicina Traslacional (IATIMET), Universidad de Buenos Aires, Marcelo T. de Alvear 2270, Ciudad Autónoma de Buenos Aires C1122AAJ, Argentina; rcastillalozano@fmed.uba.ar; 4 Consejo Nacional de Investigaciones Científicas y Técnicas (CONICET), Godoy Cruz 2290, Ciudad Autónoma de Buenos Aires C1425FQB, Argentina

**Keywords:** ischemia–reperfusion, antidiarrheal, antispasmodic, flavonoids, terpenes

## Abstract

*Salvia guaranitica* A.St.-Hil. Ex Benth. (Lamiaceae) is native from South America, traditionally used for releasing tension and treating gastrointestinal cramps. It was evaluated for the presence of flavonoids and isoflavones in the extract, and the hypothesis of being cardioprotective under ischemia and reperfusion (I/R), antidiarrheal, antispasmodic and anxyolitic in respective models. For phytochemistry, GC-MS and HPLC-DAD methodologies were used. Cardiac performance after subchronic oral administration of *S. guaranitica* tincture (S.g-T, 0.95 mg extract/mL) was evaluated in rat isolated perfused hearts exposed to I/R, and mechanisms were characterized. Antispasmodic effects of S.g-T were evaluated on contractile concentration–response curves (CRCs) of carbachol (CCh) and calcium (Ca^2+^) in rat isolated intestinal tissue. In vivo tests of ricin-oil diarrhea models, open-field (OFT), novel-suppressed feeding (NFT) and tail suspension (TST) tests were performed on mice. Phenolic acids, genistin, luteolin, kaempferol and quercetin glycosides, genins, loliolide, dodecane, farnesene, phytol and isochiapin B were identified. Post-ischemic cardiac recovery was improved by S.g.-T, associated to activation of phosphatidylinositol-3-kinase and β-estrogenic receptor. S.g.-T also induced antispasmodic effects, as a non-competitive inhibitor of the CCh-CRC and Ca^2+^-CRC in the intestine. In vivo, S.g.-T showed antidiarrheal activity (95 mg extract/kg) and anxiolytic-like effects (0.95–9.5 mg extract/kg) without sedation or antidepressant-like effects. Therefore, *S. guaranitica* leaves have potential therapeutic cardioprotective, antidiarrheal, antispasmodic and anxiolytic effects.

## 1. Introduction

The genus *Salvia* (Lamiaceae) has more than 900 species distributed in the world, with several medicinal uses. These plants have a high content of polyphenols, such as flavonoids, antocyanines and antocyanidines. These types of compounds have medicinal applications, such as in the root of *Salvia milthiorriza*, effective in cardiovascular, renal and hepatic diseases [1]. In fact, *S. miltiorrhiza* root (danshen) has demonstrated its effectiveness in reducing cardiac infarct size and mortality in rats, attributed to the presence of phenolic acids such as salvianolic acids as the main active components of the aqueous extract, with antioxidant effects [2]. Another internationally used species is *Salvia officinalis* L., for treating dysmenorrhea, because leaf extracts showed emenagogue, estrogenic and antispasmodic properties [3]. Accordingly, the activity of *S. officinalis* was experimentally demonstrated as antidiarrheal and antispasmodic [4].

In South America, there is a native plant, *Salvia guaranitica* A.St.-Hil. Ex Benth., which grows in Paraguay, South of Brazil, Uruguay and the Argentinian northeast. It has several synonimia, such as *S. coerulea* Benth., *S. ambigens* Briq., and *S. melanocalyx* Briq. [5]. Traditional applications of *S. guaranitica* for releasing tension and treating gastrointestinal cramps are known. Previous reports showed the presence of flavonoids and phenylpropanoids, such as kaempferol and cirsiliol, after fractioning the hydroethanolic extract of leaves with diethyl ether and with preparative chromatography [6]. Cirsiliol was able to bind the benzodiazepine receptor and produce sedation without anxyolitic effects [6,7]. Moreover, the terpenic composition of the essential oil of *S. guaranitica* was studied [8]. However, the scientific phytochemical and biological basis of medical applications had not been examined in the popularly used hydroethanolic extract of this plant. Then, considering the presence of flavonoids in this and other species of *Salvia* as secondary metabolites of the plant, we hypothesized their presence in the hydroethanolic extracts of *S. guaranitica*, as well as terpens provided from partial dissolution of the essential oil of this aromatic plant. It is known that both types of metabolites, flavonoids and terpenes, could confer biological activities such ischemic cardioprotection, antispasmodic and anxyolitic effects.

Although previous studies focused on isolated compounds or the composition of the essential oil, an integrated phytochemical and pharmacological evaluation of the traditionally used hydroethanolic extract has not been reported. Therefore, the present study was designed to combine the chemical characterization of this extract with the evaluation of its biological activities and underlying mechanisms, providing experimental support for its traditional medicinal use and contributing to the knowledge of plant-derived bioactive natural products.

The aim of this experimental work was to evaluate the presence of flavonoids and terpens in the hydroethanolic extract of *S. guaranitica* leaves and the biological activities underlying the traditional uses in the gastrointestinal system, behavior and potential cardioprotection in a model of ischemia/reperfusion after oral treatment, as well as the respective mechanisms of action.

## 2. Results

### 2.1. Phytochemical Profiles of the Extract

Preliminary analysis of the hydroethanolic extract revealed a complex metabolic composition by thin-layer chromatography (TLC), highlighting terpenic and phenolic derivatives. Regarding phenols, the TLC profile showed caffeic (Rf: 0.89) and chlorogenic (Rf: 0.46) acids, genistin (Rf: 0.63), luteolin glycosides (Rf: 0.60), apigenin glycosides (Rf: 0.74), kaempferol glycosides (Rf: 0.95), and traces of luteolin, kaempferol, and quercetin (Rf: 0.96). In order to delve deeper into the study of the chemical composition and analyze the relationship with the pharmacological properties evidenced, the chloroform and ethyl acetate fractions were obtained from the hydroethanolic extract (see point 4.3).

The total flavonoid content was 23 ± 0.69 and 83.6 ± 0.7 mg/g for the hydroethanolic extract at 70% (tincture) and the ethyl acetate fractions.

**Figure 1:**
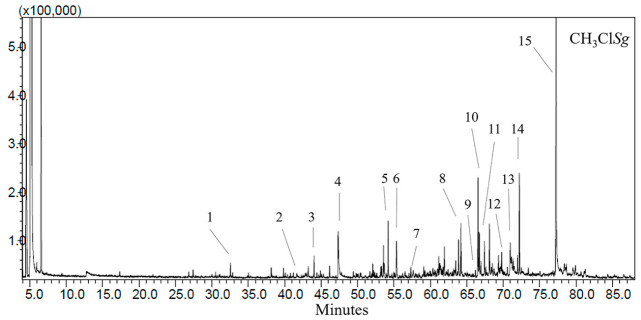
Chromatogram of the chloroform fraction obtained from the hydroethanolic extract of leaves of *S. guaranitica* (Lamiaceae), by GC-MS. Signal numbers are explained in Table 1.

In the chloroform fraction, several metabolites were identified by gas chromatography with mass spectrum detection (GC-MS) (Figure 1). Phytol (diterpene alcohol, trans and isomer), ethyl palmitate, isochiapin B (sesquiterpene lactone), (-)-loliolide (monoterpene lactone), spathulenol and farnesane (sesquiterpene) were highlighted (Table 1).

**Figure 2:**
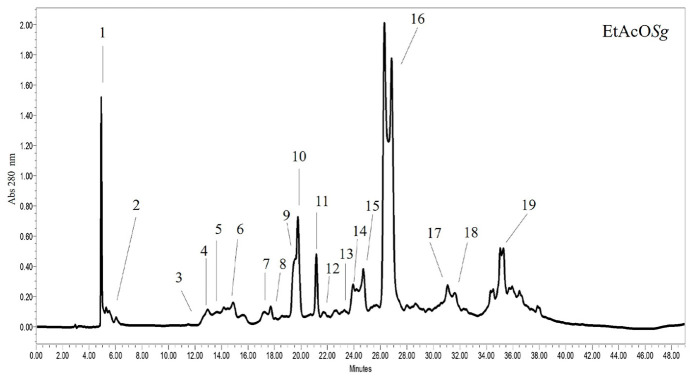
Chromatographic profiling of ethyl-acetate (EtAcO) fraction from the hydroethanolic extract obtained from *S. guaranitica* (Lamiaceae) leaves for HPLC-DAD. Signal numbers are explained in Table 2.

The phenolic profile was obtained by high-performance liquid chromatography with a photodiode array detector (HPLC-DAD) from the ethyl acetate (EtAcO) fraction (Figure 2). Phenolic acids were identified, highlighting gallic, protocatechuic, chlorogenic, caffeic and hydroxybenzoic derivatives; salvianolic acid was also possibly identified. Regarding flavonoids, glycosylated derivatives of apigenin, luteolin, and kaempferol were prominent, as well as methoxylated derivatives, apigenin, naringenin, and remnants of genistin and penta-O-methylquercetin (Table 2).

### 2.2. Cardioprotection of S. guaranitica in Ischemia/Reperfusion

**Figure 3:**
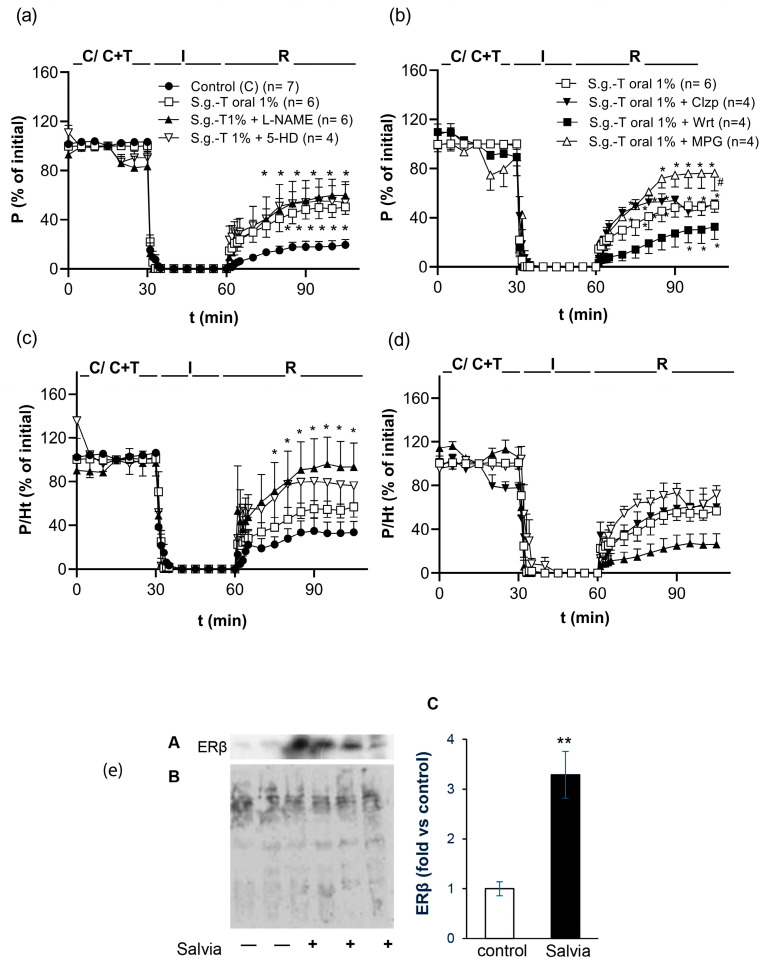
Effects of oral subacute administration of *S. guaranitica* 1% *w*/*v* hydroethanolic extract (tincture, S.g-T) on the rat isolated ventricles exposed to ischemia/reperfusion and evaluation of the underlying mechanisms by perfusing L-NAME, 5-HD, clonazepam (Clzp), wortmannin (Wrt) and MPG. In (**a**,**c**): Maximal contractile pressure developed (P) as % of initial control. In (**b**,**d**): Total muscle economy (P/Ht) as % of initial control. * *p* < 0.05 vs. control, # *p* < 0.05 vs. S.g.-T (two-way ANOVA results in Table A2). In (**e**): ERβ expression determined by Western blot. (A) Representative blot from three independent experiments. (B) PVDF membrane stained with Ponceau S, used as a loading control. (C) ERβ band intensities were normalized to Ponceau staining and expressed as fold change relative to control. Data are presented as mean ± SEM. *p* < 0.01, ** *p* < 0.001.

The initial parameters of isolated hearts, either contractile (P as maximal pressure developed) and energetic (Ht as total heat production, and P/Ht as total muscle economy), are shown in Table A1. Figure 3 shows that subacute administration of the hydroethanolic extract of *S. guaranitica* (prepared as a tincture, S.g-T) diluted to 1% *w*/*v* leaves during 7 days (equivalent to oral 105 mg extract/kg/day) improved the contractile post-ischemic recovery of hearts (Figure 3a). At the end of the period of 45 min reperfusion, non-treated control hearts recovered 19.5 ± 4.2% of initial P (n = 7), while in the hearts treated with S.g.-T it increased to 50.3 ± 6.0% of initial P (n = 6) (Figure 3a). After measuring Ht and calculating muscle economy as the ratio P/Ht, this one was also improved in hearts from rats treated with the extract (Figure 3b). However, Table 3 shows that the extract treatment did not modify the typical diastolic contracture produced during I/R.

Looking for the mechanism responsible for cardioprotection, neither the blockade of the mitochondrial ATP-dependent potassium channels (mKATP) with 5-hydroxi-decanoate sodium (5-HD) nor the blockade of nitric oxide-synthases (NOSes) with Nω-Nitro-L-arginine methyl ester hydrochloride (L-NAME) before I/R changed the post-ischemic protection in P, although L-NAME increased the economy P/Ht (Figure 3a,b). Moreover, Figure 3c,d show that blockade of the mitochondrial sodium–calcium exchanger (mNCX) with clonazepam (Clzp) did not change the post-ischemic recovery either. However, blockade of phosphatidylinositol-3-kinase (PI3K) activation with wortmannin before I/R reduced the post-ischemic recovery of P and P/Ht (Figure 3c,d) and significantly increased the diastolic contracture (ΔLVEDP) during I/R (Table 3), concluding that this pathway is involved in cardioprotection. In another way, perfusion of hearts with the scavenger of the reactive oxygen species (ROS) called N-2-mercaptopropionylglicine (MPG) before I and during R improved still more the recovery of hearts, without changing the economy P/Ht, suggesting that *S. guaranitica* did not totally prevent ROS accumulation during I/R (Figure 3c,d). Table A2 shows the statistical analysis of all mechano-energetic protocols. Moreover, reperfused hearts from rats orally treated with S.g.-T 105 mg extract/kg/day showed significantly higher amounts of beta-estrogenic receptor than non-treated rats’ reperfused hearts, as shown in the Western blot results in Figure 3e.

### 2.3. Antispasmodic Effects of S. guaranitica

**Figure 4:**
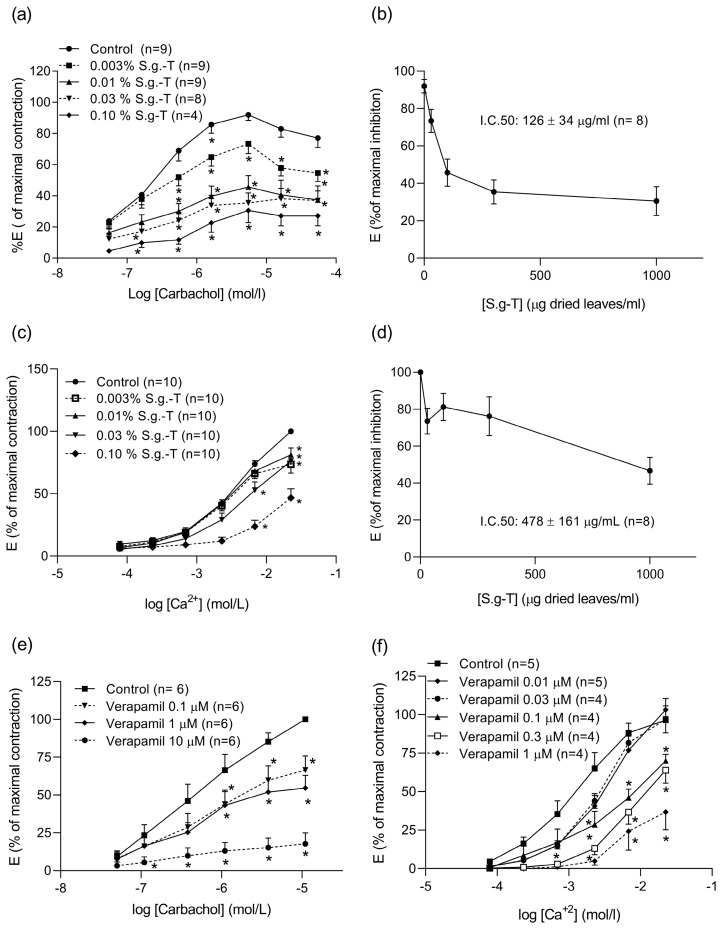
Effects of the hydroethanolic extract of *S. guaranitica* (tincture, S.g.-T) on rat intestinal concentration–response curves of carbachol (CCh-CRC, (**a**)) and calcium (Ca^2+^-CRC, (**c**)). In (**b**,**d**) are the respective inhibition curves for calculation of IC50. In (**e**,**f**): Effects of verapamil (as positive control) on the CRCs of carbachol (**e**) and calcium (**f**). * *p* < 0.05 vs. control (two-way ANOVA results in Table A3).

In isolated rat intestines, the hydroethanolic extract of *S. guaranitica* inhibited the contractile responses during the CCh-CRCs as a non-competitive antagonist (Figure 4a). The −log (50% effective concentration) (pCE50) of carbachol was 6.66 ± 0.17, and the 50 inhibitory concentration (IC50) of the extract resulted in 126.3 ± 34.3 µg leaves/mL (Figure 4b). Considering that the yield was 9.5 g extract/100 g leaves, the IC50 was equivalent to 12 µg extract/mL. When the extract was assessed on Ca^2+^-CRCs in depolarizing solution in order to evaluate whether it affects Ca^2+^ channel influx, it inhibited the Ca^2+^-CRCs in a non-competitive way (Figure 4c). The inhibition curve shows that IC50 was 478 ± 161 µg leaves/mL (Figure 4d), equivalent to 45 µg extract/mL. The non-competitive effects of *S. guaranitica* were similar to those of verapamil, which was assessed as a positive control (Figure 4e,f). Table A3 shows the statistical analysis of all the protocols.

### 2.4. Effects of S. guaranitica on the Diarrhea Model

**Figure 5:**
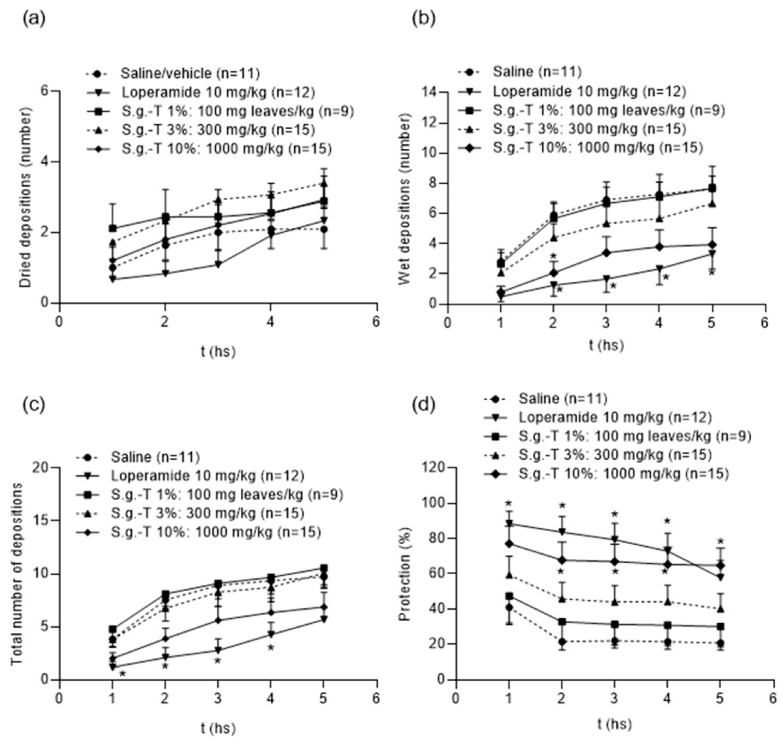
Effects of the hydroethanolic extract of *S. guaranitica* (tincture, S.g.-T) on the murine model of diarrhea induced by castor oil in mice over 5 h. (**a**) Number of dried depositions. (**b**) Number of wet depositions. (**c**) Total number of depositions. (**d**) Degree of protection (% of dried/total). Results are expressed as mean ± SEM (n = 8). Data were analyzed by two-way ANOVA; significant post hoc comparisons (Tukey’s test) are indicated (* *p* < 0.05 vs. saline) (two-way ANOVA results in Table A4).

The oral administration of the extract of *S. guaranitica* at doses of 1000 mg leaves/kg or 95 mg extract/kg (n = 15) significantly reduced the number of wet depositions of mice treated 1 h later with ricin oil after 2 h (Figure 5). Lower doses had no significant effect, but the effect was comparable with that of loperamide 10 mg/kg (n = 12) as positive control (Figure 5). Number of dried depositions was not significantly changed (Figure 5), but the % of protection was significantly increased at the doses of 95 mg extract/kg, as well as loperamide (Figure 5d). Table A4 shows the statistical analysis of all the parameters.

### 2.5. Effects of S. guaranitica on Behavior Mice Tests

**Figure 6:**
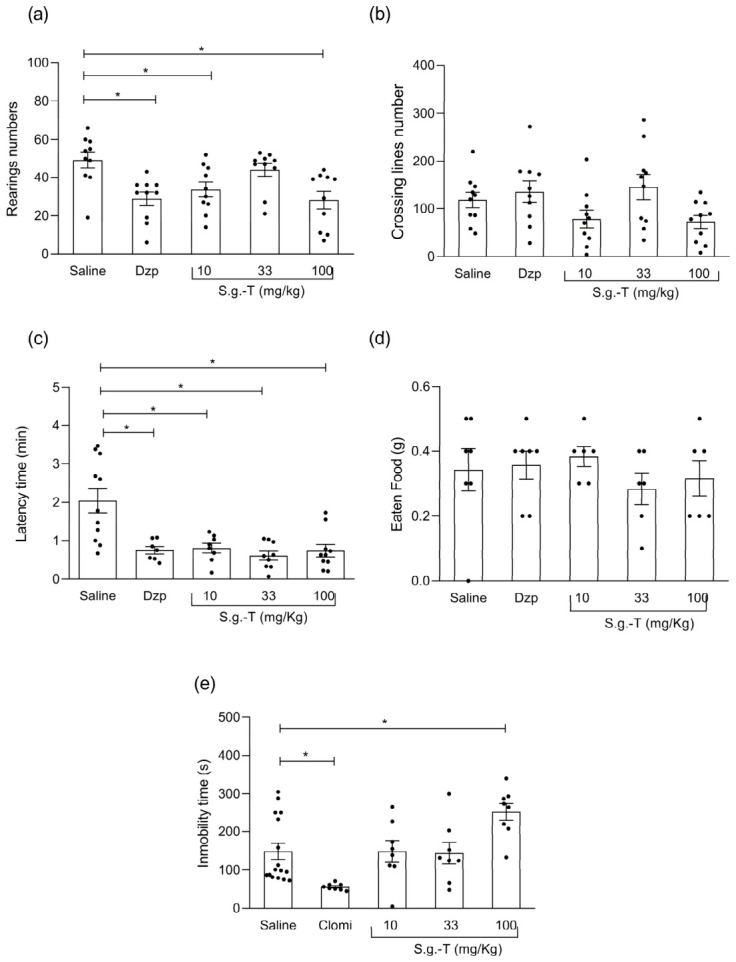
Effects of *S. guaranitica* leaf hydroethanolic extract (tincture, S.g-T) on the behavioral tests in mice. In the open-field test (OFT): number of rearings (**a**) and crossed lines (**b**). In the novelty suppressed feeding test (NFT): latency time (**c**) and eaten food (**d**). In the tail suspension test (TST): immobility time (**e**). Post-tests: * *p* < 0.05 vs. control. (two-way ANOVA results in Table A5).

The hydroethanolic extract of *S. guaranitica* (tincture, S.g-T) reduced spontaneous exploration in the open-field test (OFT) at i.p. doses of 10 and 100 mg leaves/kg (0.95 and 9.5 mg extract/kg) as well as the positive control diazepam at 1 mg/kg, doses in which they did not significantly change locomotion (Figure 6a,b, n = 10). However, in the food suppression novelty test (FNT) S.g-T via i.p. at all doses of 10, 33 and 100 mg leaves/kg significantly reduce the latency time, as well as diazepam at anxiolytic doses as positive control (0.3 mg/kg), with respect to the negative control (saline with ethanol) (Figure 6c, n = 7–10). Moreover, the possibility that changes were a response to orexigenic effects was rejected because neither of the groups altered the amount of food eaten (Figure 6d). Results suggest an anxiolytic-like effect.

However, when assessed, for *S. guaranitica* extract in the tail suspension test (TST) in comparison to clomipramine, there was no reduction of the immobility time in any of the evaluated doses from 10 to 100 mg leaves/kg (Figure 6e). Results suggest that this plant does not have a potential antidepressant-like effect. Table A5 shows the statistical analysis of all the behavioral tests employed.

## 3. Discussion

This work describes for the first time an integral phytochemical and biological study of the hydroethanolic extract of *S. guaranitica*, that is, in the more commonly used extract of this native plant from South America. We have shown the presence of several phenols, such as flavonoids and phenolic acids, and terpenes in the extract. Moreover, we have reported the gastrointestinal and cardioprotective effects of the hydroethanolic extract. Results demonstrating antidiarrheal and antispasmodic effects support the applications of this plant as a traditional use for treating gastrointestinal disorders. Moreover, the cardioprotection against ischemia after oral administration of the extract to rats during 1 week suggests a new application of the extract of *S. guaranitica*. The main mechanisms associated with those effects were described. Regarding behavioral applications, the hydroethanolic extract demonstrated anxiolytic activity but not sedative nor antidepressant effects.

By oral administration, the extract of *S. guaranitica* at 1 mg leaves/kg (95 mg extract/kg) reduced the number of wet depositions, with a relative increase in dried depositions with respect to total number, in a way that significantly increased the protection degree against diarrhea induced by ricin oil. This effect was not different to that of loperamide as positive control, although this one reduced the total number of depositions more. The ricinoleic acid of castor oil is a known activator of contraction in the colon, while the µ–agonist loperamide presynaptically reduces acethylcholine release and consequently attenuates peristaltism. The differences in the antidiarrheal properties of the extract (S.g.-T) and loperamide suggest beneficial effects of *S. guaranitica*, affecting the normal peristaltism less than loperamide. In comparison, the 70% protection against diarrhea seen in S.g.-T at 95 mg extract/kg was similar to the effect shown by 300 mg extract/kg of *Matricaria chamomilla*, which was due to activation of K^+^ channels (voltage-dependent and ATP-dependent), and Ca^2+^-channel inhibition [9]. Moreover, the phosphodiesterase-inhibition contributes to the spasmolytic effect of *M. chamomilla* [10]. This plant is rich in flavonoids and terpenes, such as bisabolol, apigenin-7-glycoside, luteolin and quercetin glycosides [11], some of them present in *S. guaranitica*. On the other side, *S. officinalis* also exhibits antidiarrheal activity, with antisecretory and astringent effects mediated by phenolic acids, flavonoids, and tannins, as well as antioxidant and anti-inflammatory effects to protect mucosa [12]. These compounds present in the *Salvia* genus could contribute to the effect of *S. guaranitica*. The total flavonoid content in the extract of *S. guaranitica* (23 mg/g) was comparable to those described in other *Salvia* species, e.g., *S. fruticosa, S. officinalis* and *S. verticillata* (31, 37 and 39 mg/g, respectively) [13]. Similarly, the hydroethanolic extract of *S. guaranitica* reduced intestinal contractility in the ex vivo carbachol CRCs. It inhibited the contraction induced by carbachol in a non-competitive way, with an IC50 value of 12 µg extract/mL. The mechanism involved the inhibition of Ca^2+^ influx to the smooth muscle, with a non-competitive blockade, which was similar to that of the known Ca^2+^-channel blocker verapamil [14,15]. Taken together, the complex mixture of flavonoids and phenolic compounds in *S. guaranitica* would modulate the intestinal contractility and Ca^2+^ influx, thus producing an effective reduction of diarrheal symptoms without compromising normal peristaltic function.

The presence of phenolic acids and flavonoids in *S. guaranitica* was demonstrated by HPLC-DAD, including gallic, chlorogenic, caffeic and salvianolic acids, apigenin and luteolin glycosides (as described in *M. chamomilla*), kaempferol glycoside, vitexin and methyl flavonoids. The most abundant compounds identified in the extract were tricetin-3′,4′,5′-trimethylether, apigenin-7-O-glucoside, scutellarein-6,4′-dimethylether, scaposin, 7-O-methylapigenin, gallic acid and luteolin-3′-O-glucoside. Some of these compounds were reported as responsible for antispasmodic effects in intestinal smooth muscles. In particular, some of them interfere with the Ca^2+^ influx to the rat intestine, such as vitexin [15], kaempferol and others [16,17,18,19]. Recently, it was reported that an extract of *Vaccinum myrtillius* leaves is rich in chlorogenic acid and is an intestinal spasmolytic by non-competitive inhibition of agonist-CRCs as acethylcholine and histamine and inhibition of Ca^2+^ influx [20]. Therefore, our results about antispasmodic and antidiarrheal effects of *S. guaranitica* extract agree with the phenolic profile and with the previously reported properties of those phenolic compounds.

In addition, many of the phenolic compounds identified in the EtAcO fraction, such as chlorogenic acid, quercetin, apigenin, salvianolic acid, scutellarein-6,4′-dimethylether and others related, were described as antioxidant and preventive of cardiovascular and intestinal diseases [21,22,23,24]. Moreover, despite that the isoflavone genistin was found in low amounts in the extract, genistein was reported as cardioprotective when administered as a pure compound [25]. As hypothesized from composition, the oral administration of *S. guaranitica* extract to rats during one week (doses of 105 mg extract/kg/day) prevented the post-ischemic dysfunction in isolated hearts. In fact, it increased the contractile post-ischemic recovery to 50% of initial P after 45 min reperfusion in comparison to 20% in non-treated rat hearts, and the same for total muscle economy (P/Ht). Looking for the mechanism of action responsible of cardioprotection, pretreatment with L-NAME, 5-HD and clonazepam did not significantly modify the recovery of hearts from rats treated with S.g-T (Figure 3), suggesting that the plant would not activate NO-synthases, mitochondrial KATP-channels nor mitochondrial Na/Ca-exchanger (mNCX). Contrarily, these mechanisms were described in cardioprotection of isoflavones because their activation reduces the mitochondrial Ca^2+^ overload, which becomes apoptosis or cardiac stunning [24]. However, the perfusion of isolated hearts with wortmannin, which inhibits the activation of phosphatidylinositol-3-kinase (PI3K), significantly reduced the post-ischemic recovery of hearts from S.g.-T-treated rats, suggesting that this pathway was activated by the oral treatment. In fact, the activated form of PI3K interacts with the kinase Akt to initiate a fall of events of survival mechanisms in the heart, which attenuates apoptosis and regulates glycogen synthesis and glucose transport [26]. Wortmannin inhibits the Akt phosphorylation induced by PI3K, and then it reversed the reduction in infarct size induced by the ischemic preconditioning [27]. Moreover, cardioprotection develops in mitochondrial functional preservation, delaying the Ca^+2^-induced mPTP opening, and so maintaining myocardial viability [28]. Therefore, subchronic administration of the hydroethanolic extract of *S. guaranitica* developed cardioprotection with a mechanism similar to that of preconditioning, with preservation of mitochondrial metabolism. These beneficial effects could be mediated by the presence of antioxidants components in the extract, such as the mentioned flavonoids and phenolic acids [21,23]. In addition, reperfused hearts of rats orally treated with S.g.-T showed an increase in the expression of beta-estrogenic receptors (βERs) with respect to non-treated reperfused hearts, suggesting that flavonoids and the isoflavone genistin could increase the estrogenic cardioprotection. It has been reported that activation of the βERs by estrogens induces activation of PI3K as a cardioprotective mechanism during I/R [29]. Therefore, our results agree with a phytoestrogenic effect of certain components of the extract. In fact, it was reported that some phytoestrogens such as genistein, apigenin, naringenin, and kaempferol compete more with estradiol for binding to βERs than to alpha-ERs [30].

In addition to phenolic derivatives, other terpenics were identified in the chloroformic fraction of the hydroalcoholic extract by GC-MS. Terpenes have demonstrated various pharmacological properties, among them those related with this study, gastrointestinal and cardioprotective [31]. The phytol isomer showed to be the most abundant in the extract, followed by ethyl palmitate, trans-phytol, isobenzofuranone and 2,4-ditert-butylphenol. In other species, e.g., *S. dichroantha*, phytol is a major component (33.27%) [32]. In agreement with the effect of *S. guaranitica* extract, phytol is a chlorophyl-derived diterpenoid with antioxidant and antidiarrheal effects on castor oil-induced diarrhea [33]. The essential oil of *S. guaranitica* contains 60% of sesquiterpene hydrocarbons (such as germacrene D and β-caryophyllene) and 4% of monoterpenes [8]. In agreement, the *S. guaranitica* hydroethanolic extract had other monoterpenes and sesquiterpenes, such as isochiapin B, (-)-loliolide, spathulenol, and farnesane. Loliolide has shown mitochondrial protection, reduction of oxidative stress and apoptosis [34]. Then, terpens could also contribute to cardioprotection. Moreover, many of the components identified in *S. guaranitica* agree with the findings of Afzal et al. [35], who described the chemical composition and pharmacological activity of several other species of *Salvia*, highlighting phenolic acids, flavonoids and terpenes.

On the other hand, the extract of *S. guaranitica* was demonstrated to reduce mice explorative spontaneous behavior with respect to the vehicle in the open field test (OFT) without affecting spontaneous locomotion. The anxiolytic effect was then confirmed in the novelty feeding suppressed test (NFT) because S.g.-T at 0.95 to 9.5 mg extract/kg reduced the latency time to feed. The fact that they did not change the amount of food eaten lets us exclude the effect as orexigenic. These results are compatible with a mild central inhibitory or anxiolytic-like effect rather than a nonspecific motor or motivational impairment. Thus, probably due to low concentration, the whole hydroethanolic extract did not show the sedative effect previously found in the fractions enriched in cirsiliol at a higher dose (3 g leaves/kg) [7]. However, the anxyolitic effects agree with the previous characterization of components that bind to benzodiazepine receptors [6]. The intraperitoneal doses used are more than 10 times lower than those orally applied to obtain an antidiarrheal effect, and so it could be expected that this central inhibition could contribute to the therapeutic intestinal effects. The central effect may be at least partly attributed to the flavone derivatives apigenin and luteolin, to the flavonol kaempferol and to the methylated flavonoids identified in this work, as it was reported for other plants [36,37]. Moreover, phytol has been described as having anxiolytic and antidepressant properties [38]. However, when assessing the *S. guaranitica* extract in the tail suspension test (TST) in comparison to clomipramine, there was no reduction of immobility time in any of the evaluated doses, suggesting that the extract does not produce antidepressant-like effects.

The behavioral findings are also consistent with previous reports in several species of the genus *Salvia*, which depend on the species, dose and experimental paradigm. Antidepressant-like activity has been described for *S. verticillata*, *S. elegans* and *S. officinalis* in rodent models without inducing motor dysfunction [12,39,40,41,42]. Sedative effects have been reported for *S. divinorum* and *S. miltiorrhiza* [43,44]. In contrast, under the experimental conditions evaluated, the *S. guaranitica* extract only produced a mild central inhibitory or anxiolytic-like effect. There is a functional interaction between central regulatory mechanisms and gastrointestinal motility [9], and the ethnopharmacological uses of the genus *Salvia* include the treatment of nervous and gastrointestinal disorders [39]. Therefore, our results suggest that the anxiolysis observed for *S. guaranitica* could partially contribute to its antispasmodic and antidiarrheal activities.

## 4. Materials and Methods

### 4.1. Chemicals and Reagents

Standard flavonoids of HPLC quality were from Carl Roth (Karlsruhe, Denmark). C8-C40 Alkanes Calibration Standard Supelco (Bellefonte, PA, USA) was used for determine retention times. Drugs employed in biological tests included: Carbamylcholine chloride (carbachol, CCh, Sigma-Aldrich, St Louis, MO, USA), ω-Nitro-L-arginine methyl ester hydrochloride (L-NAME, Sigma-Aldrich, St Louis, MO, USA), 5-hydroxydecanoate sodium salt (5-HD, Sigma-Aldrich, St Louis, MO, USA), wortmannin (Wrt, Sigma-Aldrich, St Louis, MO, USA), N-(-2-mercaptopropionil)-glycine (MPG, Sigma-Aldrich, St Louis, MO, USA), clonazepam and diazepam (Saporiti, Buenos Aires, Argentina). Tyrode’s solution was prepared with (in mmol/L) 150 NaCl, 2.7 KCl, 2 MgCl_2_, 12 NaHCO_3_, 0.4 NaH_2_PO_4_, 1.8 CaCl_2_ bubbled with air (pH 8.2). Ca^2+^-free solution (Tyrode’s-0Ca^2+^) was obtained avoiding addition of CaCl_2_ to Tyrode solution, and Tyrode-0Ca^2+^-40 mM K^+^ was obtained by adding 10% KCl (0.6 mL) to 20 mL Tyrode’s 0Ca^2+^, as previously described [14]. Krebs solution contained (in mmol/L) 1 MgCl_2_, 125 NaCl, 0.5 NaH_2_PO_4_, 7 KCl, 25 NaHCO_3_, 2 CaCl_2_ and 6 dextrose, bubbled with 95% O_2_^−^ 5% CO_2_, as previously reported [24].

### 4.2. Plant Material and Extracts

Leaves and flowers of *S. guaranitica* variety “argentine sky”, by the clear blue color of flowers, were collected in La Plata city, Gonnet neighborhood (34°52′ S, 57°54′ W) in January 2019. Aerial parts were allowed to dry at room temperature for 4 weeks and identified by M.Sc. Agr. Eng. Marta Colares in the Herbarium (LPAG) from Universidad Nacional de La Plata. The hydroethanolic extract (prepared as a tincture, S.g-T) was obtained by maceration at 10% *w*/*v* of dried leaves (herbal drug) in ethanol 70° for at least 48 h, accordingly to Farmacopea Argentina (Argentinian Pharmacopoeia). The yield of dried extract was calculated by evaporation (9.5 ± 2.0 g extract/100 g leaves). The medium yield was used to convert the IC50 of the tincture, initially expressed as % *w*/*v* of leaves in μg extract/mL, and the doses in mg extract/kg.

For the biological ex vivo studies, the hydroethanolic extract (tincture, S.g-T) was diluted in Tyrode’s solution on the day of the experiment (to 0.003, 0.01, 0.03, and 0.1% leaves *w*/*v*), and their concentrations were expressed as μg extract/mL. Negative controls with vehicle (70° ethanol 0.1% *v*/*v*) were evaluated. For in vivo experiments, the extract (10% *w*/*v*) was diluted (to 0.3, 1 and 3% *w*/*v*), and doses were expressed in mg extract/kg.

### 4.3. Analysis of Flavonoids in the Hydroethanolic Extract

Dried powdered leaves of *S. guaranitica* A. St.-Hil. ex Benth. were extracted with ethanol 70° at room temperature twice for 48 h each time. After that, the extracts were separated by centrifugation at 4 °C, the solvent was removed by a rotary evaporator and in vacuum oven. Planar and HPLC chromatographic profiles of the hydroethanolic extracts were performed. One part of the first hydroethanolic extract was suspended in water, and then partitioned between chloroform and ethyl acetate, successively. TLC was developed on silica gel G60 F254 plates with mobile phase of ethyl acetate–formic acid–glacial acetic acid–water (100:11:11:26) and revealed with Natural Products—Polyethylene Glycol (NP-PEG) at 365 nm UV light. The Rf values were measured and compared with standards and bibliography for the identification of the components.

For the determination of the total flavonoid content, the previously described technique was used with modifications [45]. Aliquots of 0.1 mL extract were mixed with 1.4 mL of distilled water and 0.5 mL of the reagent (133 mg AlCl_3_, 400 mg sodium acetate in 100 mL of a solvent consisting of 140 mL methanol, 50 mL water, and 10 mL acetic acid). After 30 min at room temperature, the absorbance was measured at 415 nm. For quantitative analysis rutin was chosen as the reference compound. Flavonoid content is expressed as mg rutin/g dry material. All measurements were performed in triplicate.

### 4.4. Gas Chromatography with Mass Spectrum Detection (GC-MS)

Chloroform fraction was analyzed by GC-MS. The GC-MS analysis was carried out with a Shimadzu GCMS–QP2010 system (Shimadzu Corporation, Kyoto, Japan). Mass spectra were recorded at 70 eV. The mass range was from *m*/*z* 35 to 450. An aliquot of 1 µL chloroform fraction solution (10 mg/mL in chloroform) was injected. A SPB-5 column 5% diphenyl/95% dimethyl polysiloxane (60 m × 0.25 mm, 0.25 mm film thickness) was used with helium as the carrier gas (1.0 mL/min). The GC oven temperature was kept at 40 °C for 10 min and programmed to 200 °C at a rate of 3 °C/min, then to 220 °C at a rate of 2 °C/min, and then was kept at 220 °C for 15 min. The injector temperature was set at 200 °C. Split was the injection method used. The interface temperature was 280 °C. Detection was performed by comparing the mass spectra of the detected substances with those contained in the Wiley 9 library available to the equipment [43] (with modifications). The proportion of constituents identified as area % (A %) was also determined, as well as the Linear Retention Indices (RIs) [46] (Table 1).

### 4.5. High Performance Liquid Chromatography (HPLC-DAD)

The analysis of ethyl acetate fraction was performed in a RP-HPLC-DAD system with a photodiode array detector composed by a Waters 1525 HPLC Binary Pump, Rheodyne injector (Rohnert Park, CA, USA), Waters 2996 Photodiode Array Detector and a Waters XBridge C-18 column (3.5 μ) 150 × 3 mm (Waters Corporation, Milford, MD, USA). As mobile phase, formic acid 1% (solvent A) and acetonitrile (solvent B) were used. The elution gradient consisted of 5% solvent B and 95% solvent A for 3 min, followed by a linear gradient to 30% solvent B for 28 min. Then a linear gradient to 60% solvent B for 5 min, then 60% solvent B for 5 min, then a linear gradient to 95% solvent B for 5 min and then a sharp transition to 5% solvent B in 3 min. The chromatographic separation was performed at room temperature at a flow rate of 0.5.0 mL/min. The elution was monitored at 254 and 360 nm (spectra were obtained between 210 and 800 nm). The identification of each compound was achieved by comparing retention times and UV spectra with those of standards and bibliography [47,48] (with modifications).

### 4.6. Pharmacological Studies

#### 4.6.1. Animals

The research was conducted on both adult male and female Sprague–Dawley rats (220–270 g) for ex vivo experiments. Moreover, 2–3 month-old Swiss albino and C57BL/6 female mice (20–28 g) were used for in vivo experiments. In both species, randomization was performed to assign animals either to control or treatment protocols. Protocols followed the internationally accepted principles of laboratory animal use and care as established by US guidelines (NIH publication 85-23 revised in 1996) and principles in the Declaration of Helsinki, according to the Resolution 1047 anexo II-2005 of Consejo Nacional de Investigaciones Científicas y Técnicas (CONICET) de la República Argentina. The protocols and procedures were approved by an ethical local committee of the Facultad de Ciencias Exactas de la Universidad Nacional de La Plata (CICUAL), numbers 001-29-2018-2027 (intestinal ex vivo), 015-5-2015/2019-2025 (cardiac ex vivo), 002-47-25/2025-2031 (antidiarrheal) and 005-29-18/2018-2027 (behavior tests in mice).

#### 4.6.2. Ex Vivo Studies

##### Cardiac Preparations and Mechano-Calorimetrical Measurements

Rats (of both sexes for each treatment group) were treated during 1 week with the hydroethanolic extract of *S. guaranitica* diluted to 1:100 (1% *w*/*v* herb or 0.95 mg extract/mL) in the drinking water. The doses was calculated from the mean consumption of 10–12 mL/day/100 g b.w., being equivalent to about 1.1 mg leaves/kg/day or 105 mg extract/kg/day). After that, rats were anesthetized with an intraperitoneal injection of pentobarbital (40 mg/kg i.p.) and received heparine (2000 IU) and subcutaneous tramadol for deep analgesia (10–20 mg/kg s.c.). The heart was excised, and the coronaries were perfused by the Langendorff technique with Krebs solution, at a constant flow of 7 mL/min/g with a peristaltic pump (Gilson Minipuls 3, Villiers Le Bel, France), as previously described [24]. Atria were excised, and spontaneous beating was stopped. A latex balloon was put inside the left ventricle to measure intraventricular pressure through a cannula with water connected to a pressure transducer (Bentley DEL900, Minden, NV, USA). Perfused ventricles were introduced in the chamber of a flow calorimeter, and it was submerged in a water bath at constant temperature of 37 °C. After equilibration, the left ventricular pressure (LVP, in mmHg) and total heat rate (Ht, in volts) signals were simultaneously recorded by a PowerLab 2/26 system with LabChart A/D program (AD Instruments, New South Wales, Australia). The calorimeter is a cylindrical mass of copper that contains a chamber with 2 Peltier units (127 thermocouples each one and ceramic walls, Melchor Thermoelectrics, Trenton, NJ, USA), which have contact with the heart. The units additively record the temperature differences between the internal (heart) and external (bath) calorimetrical walls [24]. Perfused ventricles were electrically stimulated at 3 Hz (5 V, 5 ms) by means of a stimulator (Letica LE12406, Harvard Apparatus, Barcelona, Spain). Total muscle heat rate (Ht, in mW/g) was calculated from the calorimetrical signal continuously recorded in the presence of the ventricle, after subtracting the base line and multiplying for the calibration factor (in mW/v), either with or without perfusion, as previously described [24]. Maximal pressure of contraction (P) and changes in the diastolic pressure over the initial control value (ΔLVEDP) were calculated, as well as total muscle economy (P/Ht in mmHg.g/mW). For comparison among protocols, P and P/Ht were expressed as percentage of their initial absolute values (Table A3).

After stabilization, the respective perfusion protocol was developed, followed by 30 min of no-flow ischemia (I) and 45 min reperfusion (R) with Krebs solution. In order to evaluate the mechanism of action, several protocols were performed, consisting of perfusion of either Krebs solution (control) or Krebs with the following selective inhibitors 15 min before ischemia: 100 µmol/L 5-hydroxydecanoate (5HD, to inhibit mKATP channels); 100 µmol/L L-NAME (to inhibit NO-synthases); 100 μmol/L wortmannin (Wrt, to block PI3K); 10 µmol/L clonazepam (Clzp, to block mNCX), and 2 mmol/L MPG (to scavenge ROS).

##### Expression of Beta-Estrogenic Receptors

Considering the presence of isoflavones and flavonoids in the extract, it was evaluated whether the cardiac effects are related to modifications in the expression of the beta-estrogenic receptors. A group of reperfused hearts from rats treated during 1 week with the extract of *S. guaranitica* diluted 1% and another from non-treated hearts were frozen and prepared for Western blot as follows. Protein extracts from heart tissue were obtained by sonication in ice-cold RIPA buffer (50 mmol/L Tris-HCl pH 7.4, 150 mmol/L NaCl, 1% NP-40, 0.5% sodium deoxycholate, and 0.1% SDS) supplemented with a protease inhibitor cocktail (AEBSF, aprotinin, bestatin, E-64, leupeptin, and pepstatin A). Lysates were centrifuged at 20,000 rpm for 15 min at 4 °C, and supernatants were collected. Total protein concentration was determined using the Bradford assay with bovine serum albumin (BSA) as a standard. For Western blotting, 50 µg of total protein per sample were separated by a 7.5% SDS-PAGE and transferred to polyvinylidene difluoride (PVDF; Bio-Rad Laboratories Inc., Hercules, CA, USA) membranes. Transfer efficiency and equal loading were verified by Ponceau S staining. Membranes were blocked with 5% non-fat milk in Tris-buffered saline (TBS; 20 mmol/L Tris-HCl, 150 mmol/L NaCl, pH 7.6) for 1 h at room temperature, followed by overnight incubation at 4 °C with anti-ERβ primary antibody (Santa Cruz Biotechnology, Santa Cruz, CA, USA, 1:1000). After washing with TBS containing 0.1% Tween-20 (TBST), membranes were incubated for 1 h at room temperature with horseradish peroxidase (HRP)-conjugated anti-rabbit IgG secondary antibody (Santa Cruz Biotechnology, Santa Cruz, CA, USA, 1:2000). Blots were washed again with TBST and TBS and developed using an enhanced chemiluminescence (ECL) detection kit (Amersham Biosciences, Inc., Little Chalfont, UK), followed by exposure to X-ray film (Agfa Gevaert S.A., Buenos Aires, Argentina). Films were scanned, and band intensities were quantified using ImageJ 154 for Java 8 software (NIH, Bethesda, MD, USA) and normalized to the corresponding Ponceau S signal of each lane [49].

##### Intestinal Muscles Preparations and Contractile Measurements

Non-treated Sprague–Dawley rats (200–250 g) were subjected to a 14 h fasting with free access to water before experimentation. After being sacrificed using pentobarbital (60 mg/kg via i.p.), the duodenum and ileum (about 2 cm long) were excised. Intestinal tissues were individually mounted in organ chambers containing air-bubbled Tyrode’s solution (20 mL) at 37 °C [14]. All preparations were equilibrated for at least 30 min at 1 g of pre-load. Intestinal tissues were connected to isometric force transducers FORT100 from World Precision Instruments (WPI, Sarasota, FL, USA), and the signals simultaneously amplified by a 4-channel preamplifier (WPI) and recorded by a MicroDAQLite device for data acquisition system and Wave View from Eagle Technology software version 1.0.0.32 (Cape Town, South Africa). Other portions were simultaneously detected by PanLab MLT0210/A (Barcelona, Spain) isometric transducers and recorded by a PowerLab 2/26 system with LabChart A/D program (AD Instruments, New South Wales, Australia).

##### Concentration–Response Curves in Intestinal Preparations

Concentration–response curves (CRCs) of contraction in response to carbachol (CCh-CRC) were simultaneously performed as previously described, by using a negative control of vehicle and a positive control of verapamil [13]. The diluted extract was assessed at several concentrations by adding them to each preparation before the CCh-CRC in sequentially increasing concentrations. The contractile effect of each CCh concentration was expressed as percentage of the maximal contraction of tissue during the second control CCh-CRC (% Emax, percentage of maximal effect). Moreover, in order to evaluate the mechanism, another protocol was followed for assessing the extract on Ca^2+^-CRCs developed in a depolarizing solution of Tyrode-0 Ca^2+^-40 mmol/L K^+^, after the negative control of vehicle. It was also used a positive control of verapamil.

From the CRCs performed with CCh and Ca^2+^, the pEC50 (as −log EC50, in M) of the agonist was calculated. For the extract, the 50% inhibitory concentration (IC50) was calculated as previously described and expressed as µg extract/mL [14].

#### 4.6.3. In Vivo Studies

##### Model of Diarrhea

The effect of the hydroethanolic extract on a model of diarrhea in mice was investigated as previously described [8]. Briefly, 5 groups of C57BL/6 mice (eight animals each) fasted for 18 h were treated by oral cannula with saline/vehicle at 10 mL/kg (negative control group), and with loperamide at 10 mg/kg (positive control group). The experimental groups received 10 mL/kg extract diluted to concentrations of 1, 3 and 10% dried leaves/mL, which become oral doses of 100, 300 and 1000 mg dried leaves/kg (equivalent to 9.5, 28.5 and 95 mg extract/kg). One hour after those treatments, diarrhea was induced by orally administering the laxative castor oil (10 mL/kg). Mice were located in individual metabolic cages with water ad libitum for 5 h for counting the number of dried and wet feces at periods of 1 h. The percentage of protection was calculated as (number of dried feces/total feces) × 100.

##### Open Field Test (OFT)

Spontaneous locomotor and exploratory activities were evaluated in mice by using the OFT, which consisted of a white cage of 30 × 50 cm in the floor divided into 15 squares of 10 cm^2^ by black lines, and surrounded by walls 27 cm high. Adequate environmental conditions of light and temperature were maintained [50,51]. Before treatment, each mouse was placed in the same corner of the open field, allowing free exploration for 5 min. Normally fed mice were divided into 5 groups, which received i.p. injections (0.1 mL by 30 g weight or 3.3 mL/kg) of one of the following treatments: extract (diluted to 0.3, 1 and 3% *w*/*v*), vehicle (saline + ethanol 70° 3:1, as negative control), and 0.5 mg/kg diazepam (as positive control). The resulting doses of extract were 10, 33 and 100 mg dried leaves/kg or 0.95, 3.1 and 9.5 mg dried extract/kg. The test was performed 30 min after the injection of extracts or vehicle, and the number of crossed lines and the number of rearings were counted during 5 min.

##### Novelty-Suppressed Feeding Test (NFT)

Mice (fasted for 24 h with water ad libitum) were placed in individual cages, being allowed to remain for 30 min to habituate, following the method previously reported [52]. They were divided into 5 groups, which received i.p. injections (0.1 mL by 30 g weight) of one of the following treatments: ethanolic extract diluted to 0.3, 1 and 3% *w*/*v* (equivalent to 10, 33 and 100 mg leaves/kg, or respectively 0.95, 3.1 and 9.5 mg extract/kg), vehicle (saline + ethanol 70° 3:1, as negative control) and 0.3 mg/kg diazepam (as positive control). After 1 h of the i.p. administration, each animal was placed in the same corner of a 40 × 40 × 30 cm wooden box containing a white circular filter paper with a diameter of 11 cm and a single food pellet in the center. The latency time to start eating was recorded. Immediately after the first bite, each mouse was removed and placed in its individual cage with a previously weighed pellet; after 5 min the amount of food consumed was weighed. Finally, mice were removed to their home cage.

##### Tail Suspension Test (TST)

The tail suspension test (TST) was performed as previously described [53]. To assess the effects of S.g.-T, a 50 cm high, 12 cm deep, and 60 cm wide setup was used. Mice were suspended individually in separate compartments to 10 cm from the floor, with adhesive tape placed 2 cm from the base of the tail. Measurements were made 1 h after i.p. administration and videotaped for 6 min. Immobility was defined as the animal remaining in an upright position without hind limb movement. Immobility time (IT, in seconds) was recorded using a stopwatch. Clomipramine (1.25 mg/kg), a known tricyclic antidepressant, was used as the reference drug.

### 4.7. Statistical Analysis

All results are expressed as mean ± SEM (n). Statistical multiple comparisons were performed by two-way ANOVA, considering two variables influencing the effect, namely the treatment and the *x*-axis variable (agonist concentration as pCCh or pCa in CRCs or time in the cardiac and OFT experiments). In the other in vivo tests, one-way ANOVA was applied. After ANOVA, Tukey’s post hoc tests were applied for paired comparisons. Statistical analyses were performed with Graph Pad Prism 8.0 software. The significance was considered at *p* < 0.05 in all tests performed.

## 5. Conclusions

This work was orientated to study a plant native from South America that is traditionally used for medicinal applications, but that had not been previously studied in the hydroethanolic extract, that is, the traditional preparation of the plant. Previous studies had only explored the composition of the essential oil (not used for medicinal applications) and the isolated flavonoid crisiol that binds to the benzodiazepine receptor.

Accordingly, this work was designed with two focuses. One of them was to explore the phytochemical components (polyphenols and terpens) potentially responsible for the traditional uses of the *S. guaranitica* hydroethanolic extract. The second and similarly important focus was the pharmacological evaluation and mechanistic studies. Therefore, this work provides integrated experimental support for the traditional medicinal use of this plant rather than addressing these aspects separately.

This is the first study showing that the hydroethanolic extract of leaves from *Salvia guaranitica* have the important presence of flavonoids and terpens in its composition and exhibited cardioprotective, antispasmodic, antidiarrheal and anxiolytic effects. The cardioprotection against the ischemia/reperfusion dysfunction was associated with activation of the PI3K pathway and increase in the βER expression. The intestinal antispasmodic effect was a consequence of non-competitive Ca^2+^-channels blockade. The spasmolytic, antidiarrheal and anxiolytic effects give a basis to some of the traditional uses of this plant. Based on previous reports, the presence of flavonoids as flavones (apigenin and luteolin glycosides), flavonols (kaempferol glycosides, methylquercetin) and isoflavones (genistin) could be at least partially responsible for the cardioprotective, antispasmodic and anxiolytic properties. Monoterpenes and sesquiterpenes contribute to the demonstrated pharmacological properties of *S. guaranitica*. Overall, this work demonstrates that *S. guaranitica* shares with other plants of the genus *Salvia* the presence of flavonoids and terpens, as responsible for pharmacological properties. By integrating phytochemical characterization with biological and mechanistic evaluation, this study provides scientific support for the traditional medicinal use of the hydroethanolic extract and highlights its potential therapeutic application in treating anxiety, diarrhea and ischemic cardiac risks.

### Limitations

We have assessed the hydroethanolic extract because it is traditionally used in human medicine (known in pharmacy as a tincture) and generally contains a greater amount of active principles than the aqueous extract (infusion). The phytochemical study has evaluated only the profile of polyphenols and terpens, according to the hypothesis of the work. Moreover, it is a preclinical experimental study, by which conclusions were obtained in the classically used laboratory animals, rats and mice. Therefore, translation to humans would not be strictly done, since differences in pharmacokinetics, such as in absorption and metabolism, could occur. However, this work is a trigger to a potential clinical study in order to evaluate these properties in humans.

Despite these limitations, the combined phytochemical characterization and pharmacological evaluation presented here provide a basis for future studies aimed at identifying the active constituents responsible for the observed biological effects and for validating these findings in clinical settings.

## Figures and Tables

**Table 1:** Principal compounds identified in chloroform fraction of hydroethanolic extract from the leaves of *Salvia guaranitica* (Lamiaceae) for GC-MS (Rt: retention time) and their % Area. RI (Linear Retention Indices). NIST (National Institute of Standards and Technology).

Signal N°	Rt (min)	Compound	% Area	RI	RI (NIST)
1	33.5	4,5-dimethyl-nonane	1.01	943.94	1021
2	41.6	2,3-dihydrogeraniol	0.22	1200.72	1228
3	44.0	farnesane	1.61	1273.40	1286
4	47.3	1-phthalanone = 1(3H)-isobenzofuranone	5.30	1386.47	2000
5	54.2	2,4-ditert-butylphenol	4.46	1511.59	1513
6	55.3	dihydroactinidiolide	3.90	1483.85	1493
7	57.3	spathulenol	0.81	1562.97	1571
8	64.2	(−)-loliolide	3.95	1683.16	1698
9	65.8	d-nerolidol = peruviol	0.43	1602.6	1520
10	66.5	*trans*-phytol	6.31	1904	2080
11	66.9	isochiapin B	2.45	1730.01	n.d.
12	69.8	nonacosane	1.58	1002.01	n.d.
13	71.0	pentadecanoic acid	2.76	1841.61	1857
14	72.2	ethyl palmitate	8.18	1961.18	1975
15	77.3	phytol isomer	57.03	2078.63	2114

**Table 2:** Principal compounds identified in EtAcO fraction of hydroethanolic extract from the leaves of *Salvia guaranitica* (Lamiaceae) for RP-HPLC-DAD and their % Area.

Signal N°	rt (min)	λ _máx_ (nm)	Compound	% Area
1	4.9	275	gallic acid	6.47
2	5.5	259, 294	protocatechuic acid	0.22
3	11.3	255, 321	*p*-hydroxybenzoic acid + 2,5-dihydroxybenzoic acid	0.06
4	12.6	245 sh, 290 sh, 323	chlorogenic acid	0.55
5	12.9	295 sh, 323	caffeic acid	0.49
6	14.2	290 sh, 296, 322	luteolin-7,3′,4′-tri-*O*-glucuronide	0.94
7	17.2	266, 291 sh, 337	vitexin	0.55
8	17.7	254, 287 sh, 344	7,4′-di-*O*-methylflavonol	0.75
9	19.3	267, 341	apigenin-7-*O*-[rhamnosyl(1-2)glucoside]	0.51
10	19.8	267, 337	apigenin-7-*O*-glucoside	14.77
11	20.9	241, 267, 290 sh, 332	luteolin-3′-*O*-glucoside	4.53
12	21.2	267, 295 sh, 337	kaempferol-3-*O*-rhamnoside	0.80
13	23.3	267, 327	apigenin	1.51
14	23.9	287, 328 sh	naringenin	3.85
15	24.9	287, 326	scaposin (5,7,5′-trihydroxy-6,8,3′,4′-tetramethoxyflavone)	7.71
16	26.6	242 sh, 280, 330	tricetin-3′,4′,5′-trimethylether	35.69
17	30.9	268, 290 sh, 328	genkwanin = 7-*O*-methylapigenin	7.06
18	31.3	287, 323	salvianolic acid B + C	5.16
19	35.4	279, 334	scutellarein-6,4′-dimethylether	8.38

**Table 3:** Changes in the left ventricular end diastolic pressure (ΔLVEDP; in mm Hg) with respect to preischemic values, at 5 and 30 min ischemia (I) and at 5 and 45 min of reperfusion (R) in the respective protocols with *S. guaranitica* 1% *w*/*v* for a week (S.g.-T, 105 mg extract/kg/day) (Tukey post hoc tests: * *p* < 0.05 vs. control, # *p* < 0.05 vs. S.g.-T).

Experimental Group (n)	I 5′	I 30′	R 5′	R 45′
Control (n = 7)	−1.9 ± 4.1	10.7 ± 4.7	29.8 ± 7.7	21.5 ± 4.9
S.g.-T 1% (n = 6)	−0.5 ± 7.1	16.4 ± 6.6	50.1 ± 8.1	30.6 ± 8.1
S.g.-T + L-NAME (n = 6)	−5.8 ± 19.5	10.5 ± 15.7	59.1 ± 5.6	37.5 ± 6.8
S.g.-T + 5-HD (n = 4)	13.0 ± 9.6	40.9 ± 12.2	52.4 ± 16.5	26.3 ± 13.3
S.g.-T + Clzp (n = 4)	23.2 ± 7.7	36.9 ± 1.9	60.8 ± 3.1 *	46.3 ± 3.4
S.g.-T + Wrt (n = 4)	31.3 ± 6.2	61.0 ± 2.8 *^#^	94.8 ± 10.1 *^#^	87.4 ± 12.0 *^#^
S.g.-T + MPG (n = 4)	19.9 ± 8.8	41.5 ± 10.6	69.2 ± 6.7 *	45.7 ± 8.3

## Data Availability

Data supporting reported results can be found at https://ri.conicet.gov.ar/handle/11336/284061 (accessed on 27 June 2026).

## Abbreviations

The following abbreviations are used in this manuscript:

Ca^2+^	ionic calcium
CCh	carbachol
CRC	concentration–response curve
EC50	50% effective concentration
HPLC-DAD	High-performance liquid chromatography with photodiode array detector
Ht	cardiac total heat rate
LVEDP	left ventricular end diastolic pressure
LVP	left ventricular pressure
NFT	novelty-suppressed feeding test
OFT	open-field test
P	maximal pressure developed
P/Ht	total muscle economy
SEM	standard error of media
S.g.-T	tincture of Salvia guaranitica leaves (hydroethanolic extract)
TST	tail suspension test

## Appendix A

**Table A1:** Initial values of mechano-calorimetrical parameters in each experimental group of hearts before exposing to any drug and/or I/R, with statistical comparison (one-way ANOVA, * *p* < 0.05 vs. the respective non-treated control rats, ns: not significant).

Experimental Group (n)	P (mm Hg)	Ht (mW/g)	P/Ht (mmHg.g/ mW)
Control (n = 7)	99.2 ± 7.3	20.4 ± 1.5	4.9 ± 0.2
S.g.-T 1% × 7 d (n = 6)	58.2 ± 10.3	12.7 ± 2.7	4.8 ± 0.3
S.g.-T + L-NAME (n = 6)	47.1 ± 4.9 *	10.8 ± 0.9 *	5.0 ± 0.5
S.g.-T + 5-HD (n = 4)	65.5 ± 14.4	14.4 ± 2.1	4.5 ± 0.7
S.g.-T + Clzp (n = 4)	72.6 ± 7.2	14.0 ± 1.6	5.2 ± 0.3
S.g.-T + Wrt (n = 4)	61.5 ± 16.3	12.2 ± 2.7	5.0 ± 0.9
S.g.-T + MPG (n = 4)	69.8 ± 24.4	11.1 ± 3.3	6.1 ± 0.4
1-way ANOVA	F = 2.451	F = 2.954	F = 0.943
	*p* < 0.05	*p* < 0.05	*p* > 0.4, ns

**Table A2:** Statistical results of multiple comparisons for P and P/Ht among all the experimental groups of rat hearts during their exposure to I/R (shown in Figure 3). Ordinary two-way ANOVA results are shown here. Results of post-tests are shown in the respective figures.

Two-Way ANOVA	P (% of Initial)	P/Ht (% of Initial)
By treatment	F = 23.36, DF = 6 *	F = 13.06, DF = 6 *
By time	F = 2431.6, DF = 29 *	F = 61.98, DF = 29 *
Interaction	F = 2.329, DF = 174 *	F = 1.241, DF = 174 *
DF residual	840	840

* *p* < 0.0001.

**Table A3:** Statistical results of multiple comparisons for CCR obtained for all the experimental groups of rat intestines (shown in Figure 4). Ordinary two-way ANOVA results are shown here. Results of post-tests are shown in the respective figures.

Variables of 2-Way ANOVA	S.g.-T		Verapamil	
	log [CCh]	log [Ca^2+^]	log [CCh]	log [Ca^2+^]
By treatment	F = 66.3	F = 30.12	F = 38.62	F = 36.09
	DF = 4, *	DF = 4, *	DF = 3, *	DF = 5, *
By log[agonist]	F = 25.43	F = 204.1	F = 34.11	F = 153.5
	DF = 6, *	DF = 5, *	DF = 5, *	DF = 5, *
Interaction	F = 1.54	F = 4.21	F = 2.957	F = 3.793
	DF = 24, ns	DF = 20, *	DF = 15, #	DF = 25, *
DF residual	238	270	120	120

* *p* < 0.0001, # *p* < 0.01, ns: not significant.

**Table A4:** Statistical results of multiple comparisons for fecal output parameters (dried, wet, total stools and inhibition %) over a 5 h period in the castor oil-induced diarrhea mouse model (shown in Figure 5). Ordinary two-way ANOVA results are shown here. Results of post-tests are shown in the respective figures.

Variables of 2-Way ANOVA	S.g.-T and Loperamide vs. Vehicle			
	Dried	Wet	Total	Protection (%)
By treatment	F = 4.229	F = 17.37	F = 17.48	F = 24.2
	DF = 4, #	DF = 4, *	DF = 4, *	DF = 4, *
By time	F = 4.101	F = 12.26	F = 15.94	F = 2.934
	DF = 4, #	DF = 4, *	DF = 4, *	DF = 4, **
Interaction	F = 0.16	F = 0.255	F = 0.2226	F = 0.1856
	DF = 16, ns	DF = 16, ns	DF = 16, ns	DF = 16, ns
DF residual	285	285	285	285

* *p* < 0.0001, # *p* < 0.005, ** *p* < 0.05.

**Table A5:** Statistical results of multiple comparisons for the behavioral tests: open field (OFT), novelty-suppressed feeding (NFT) and tail suspension (TST) tests (shown in Figure 6). Ordinary one-way ANOVA results are shown here. Results of post-tests are shown in the respective figures.

Variables of 1-Way ANOVA	S.g.-T and Positive/Negative Controls		
	OFT	NFT	TST
	Rearings:	Latency time:	Immobility time:
	F = 5.63	F = 9.171	F = 7.382
	DF = 4, #	DF = 4, *	DF = 4, *
	Crossing lines:	Eaten food:	
	F = 2.772	F = 0.5488	
	DF = 4, **	DF = 4, ns	
DFd	45	41	43

* *p* < 0.0001, # *p* < 0.005, ** *p* < 0.05.
